# Influence of Serum Vitamin D Level in the Response of Actinic Keratosis to Photodynamic Therapy with Methylaminolevulinate

**DOI:** 10.3390/jcm9020398

**Published:** 2020-02-01

**Authors:** Ricardo Moreno, Laura Nájera, Marta Mascaraque, Ángeles Juarranz, Salvador González, Yolanda Gilaberte

**Affiliations:** 1 Dermatology Service, Hospital Univ. del Henares, Coslada, 28822 Madrid, Spain; 2 Pathology Service, Hospital Puerta de Hierro, Majadahonda, 28222 Madrid, Spain; lauranajerabotello@hotmail.com; 3 Department of Cellular Biology, Universidad Autónoma de Madrid, 28049 Madrid, Spain; 4 Medicine and Medical Specialties Department, Instituto Ramón y Cajal de Investigación Sanitaria (IRYCIS), Universidad de Alcalá, 28034 Madrid, Spain; salvagonrod@gmail.com; 5 Dermatology Service, Hospital Univ. Miguel Servet, 50009 Zaragoza, Spain; ygilaberte@gmail.com

**Keywords:** photochemotherapy, methylaminolevulinate, actinic keratosis, vitamin d, calcitriol, vitamin d receptor

## Abstract

In mouse models of squamous cell carcinoma, pre-treatment with calcitriol prior to photodynamic therapy with aminolevulinic acid (ALA) enhances tumor cell death. We have evaluated the association between vitamin D status and the response of actinic keratoses to photodynamic therapy with methylaminolevulinate. Twenty-five patients with actinic keratoses on the head received one session of photodynamic therapy with methylaminolevulinate. Biopsies were taken at baseline and six weeks after treatment. Immuno-histochemical staining was performed for VDR, P53, Ki67 and β-catenin. Basal serum 25(OH)D levels were determined. Cases with a positive histological response to treatment had significantly higher serum 25(OH)D levels (26.96 (SD 7.49) ngr/mL) than those without response (18.60 (SE 7.49) ngr/mL) (*p* = 0.05). Patients with a complete clinical response displayed lower basal VDR expression (35.71% (SD 19.88)) than partial responders (62.78% (SD 16.735)), (*p* = 0.002). Our results support a relationship between vitamin D status and the response of actinic keratoses to photodynamic therapy with methylaminolevulinate.

## 1. Introduction

Vitamin D (VD) is a prohormone involved in a wide variety of functions in the organism, and has been related with several types of cancer [1]. It has several known effects on epidermal carcinogenesis [2]: it regulates keratinocyte proliferation promoting its differentiation [3], and it prevents UV-induced mutations [4], enhancing mutation repair.

In humans, vitamin D is obtained mainly through exposure to sunlight which, in the epidermis, promotes transformation of 7-dehydrocholesterol into cholecalciferol or previtamin D3. Secondarily, cholecalciferol is hydroxylated in the liver to become 25(OH)D or calcidiol, then further hydroxylated in the kidney into 1,25(OH)D or calcitriol, the biologically active form of vitamin D [1]. Calcitriol acts on its intracellular receptor (VDR), which is present in almost all cell types in humans, and its signaling exerts antiproliferative, antiangiogenic, pro-differentiating and antiapoptotic effects [5].

Actinic keratoses (AKs) are skin areas of keratinocytic dysplasia representing a preneoplastic state—or according to some authors, an in situ form—of cutaneous squamous cell carcinoma (SCC). In AK, the severity of keratinocytic dysplasia is classified, as in other intraepidermal carcinomas (CIN for cervical, VIN for vulvar, AIN for anal intraepithelial neoplasia) into KIN (keratinocytic intraepidermal neoplasia) grade 1, 2 or 3 according to the presence of dysplastic keratinocytes in one third, two thirds or the complete thickness of the epidermis [6].

Photodynamic therapy (PDT) with aminolevulinic acid (ALA) or methyl-aminolevulinate (MAL) is effective in clearing keratinocytic dysplasia and reversing some of the molecular features of AK, such as the expression of mutant P53 [7]. In this therapy, AKs are treated with mentioned photosensitizers and exposed to specific wavelength light sources. ALA and MAL are precursors of protoporphyrin IX (PpIX), a molecule that selectively accumulates in dysplastic keratinocytes. Irradiation induces photobleaching of PpIX which is responsible for tumor cell death.

In SCC murine models, pre-treatment with topical vitamin D prior to ALA-PDT has been shown to enhance PpIX accumulation and tumor cell death [8]. This has also been observed in other rodent models with oral [9] or intraperitoneal [10] administration of calcitriol. In humans, the clinical response of AK to PDT, in a split-scalp trial comparing MAL-PDT alone vs. MAL-PDT with a pre-treatment of 15 days with topical calcipotriol (a synthetic derivative of calcitriol marketed to treat psoriasis), improved in the pretreated group [11]. Galimberti also demonstrated superior efficacy of daylight mediated MAL-PDT after pre-treatment with calcipotriol ointment [12]. 

We intended to explore if VD or its receptor play a role in the response of AK to PDT. Therefore, we designed a study to evaluate the association between the serum 25(OH)D level and the skin expression of VD receptor (VDR) in AK with the response to MAL-PDT at clinical, histological and immuno-histochemical levels.

## 2. Patients and Methods

### 2.1. Design

A prospective observational pilot study was designed to establish whether serum 25(OH)D level influences the response of AK to MAL-PDT in patients.

### 2.2. Ethics

The study was approved by the local Ethical Committee at Hospital Universitario Puerta de Hierro in Madrid (Spain). The written informed consent was obtained from all the subjects before being recruited for the study.

### 2.3. Subjects

Twenty-five patients were enrolled in the study. Inclusion criteria were as follows: having five or more neighboring AKs susceptible to be treated with MAL-PDT, located on the face or the scalp. Exclusion criteria were: unstable health conditions, such as cancer or immunosuppression; medical contraindications for the treatment, such as pregnancy or photosensitivity; allergy to the MAL or any of the excipients of the cream; and having received any treatment for face or scalp AKs within the last six months. Patients were recruited from March 2014 to September 2016. Variables such as age, gender, body mass index (BMI) and Fitzpatrick phototype were collected for each patient.

### 2.4. Treatment Protocol

Patients were treated with conventional MAL-PDT as follows. AKs were prepared for the treatment by removal of hyperkerosis through gentle use of sandpaper or curettage. Then, a 1 mm layer of MAL 160 mg/g cream (Metvix^®^, Galderma, Paris, France) was applied over each AK, spreading the remaining cream on the surrounding cancerization area. After incubation under occlusion for three hours, the whole area was exposed to a red LED device emitting at 630 nm (Aktilite^®^; PhotoCure, Oslo, Norway) with a fluence of 37 J/cm^2^. After the treatment, patients were instructed to avoid sun exposure using the same SPF 50 sunscreen cream (Heliocare Airgel, IFC, Spain) when outdoors until the end of the study.

### 2.5. Clinical Evaluation

Clinical evaluation was assessed using digital photographs before and six weeks after the treatment. Clinical lesion response was evaluated by two dermatologists who measured the reduction in the AK number and the Olsen grade in the treated area. Patient response was classified in three categories: complete response, defined by a 75% to 100% reduction in number and improvement in the grade of Olsen of the AKs; partial response when the overall improvement in number and grade was lower than 75% and higher than 25%; and improvement was lower than 25% percent.

### 2.6. Biochemical Variables

Two blood tests were performed on each patient, the first one on the day of the PDT treatment and the second one 6 weeks later, at the end of the follow-up. Serum levels of 25(OH)D (ng/mL) (electrochemiluminescence, Roche Diagnostics, Madrid, Spain) were determined in the Central Laboratory of Madrid. VD deficiency was defined as serum 25(OH)D of 20 ng/mL or less, VD insufficiency as values of 20–30 ng/mL, and sufficiency over it [13].

### 2.7. Histological and Immune-Histochemical Variables

A 3 mm punch biopsy of the index lesion (the most severe AK in the area) was performed 1 week before the treatment and 6 weeks after it. The second biopsy was taken at a minimal distance from the first biopsy scar.

The skin samples were fixed (10% formalin), embedded in paraffin, sectioned (3 µm thickness) and stained by haematoxylin and eosin, and then simultaneously subjected to immunohistochemistry using the corresponding antibodies for detection of Ki67 (prediluted; Ventana Medical Systems Tucson, AZ, USA), vitamin D receptor (VDR), P53, and β-catenin antibodies (Cell Signaling Technologies, Leiden, The Nederlands). Representative sections were examined using positive and negative controls. Immuno-histochemical evaluation of P53, Ki-67, β-catenin and VDR was performed by identifying, in each section, the area with the highest levels of immunoexpression (“hot spots”) and estimating the percentage of cells with nuclear positivity in a high-power field (×400). Intensity of VDR staining was semi-quantitatively assessed by classifying expression intensity into 4 categories: 0, absence of staining; 1, mild staining (0%–33% tumoral cell staining); 2, moderate staining (>33%–66%) and 3, intense staining (>66%–100% tumoral cell staining).

Histological and immuno-histochemical variables (histological diagnosis, histological subtype of AK, KIN grade as defined by Röwert-Huber et al. [6], β-catenin, P53, Ki67, VDR expressions and VDR intensity were evaluated by a pathologist, blind to the identity of the samples. Histological response of the AK index, assessed on hematoxylin-eosin stained sections, was defined as positive when complete clearance or at least a decrease in two KIN grades was achieved, and negative when absence of histological response or decrease of only one KIN grade was shown.

### 2.8. Statistical Analysis

Quantitative variables are expressed as mean and standard deviation (SD) and dichotomous variables as proportions. Associations between qualitative variables were assessed using Pearson’s Chi-squared test or Fisher’s exact test. Mann–Whitney U-test or Student’s t-test for paired data was used to evaluate associations between quantitative variables. Pearson correlation coefficient was calculated to evaluate the linear correlation between two variables. Statistical significance was set at *p* ≤ 0.05. Analyses were performed using SPSS Statistics (version 19.0: IBM, Armonk, NY, USA).

## 3. Results

### 3.1. Demographic Characteristics of the Sample

All twenty-five patients completed the study. However, one case was excluded from the histological analysis since the post-treatment biopsy revealed a collision of an actinic and a seborrheic keratosis. The mean age was 70.1 years (range 61–81) and 76% were males with Fitzpatrick phototype 3 (60%) or phototype 2 (40%). Most of the treated AKs were located on the scalp (64%) and 36% on the facial area (Table 1). The mean basal 25(OH)D serum levels were 25.37 (SD 9.86) ng/mL.

The severity of keratinocytic dysplasia was considered KIN3 in 7 lesions (29.17%), KIN2 in 10 (41.66%) and KIN1 in 7 (29.17%) AK.

### 3.2. Clinical and Histological Response to PDT Per Lesion

**Figure 1:**
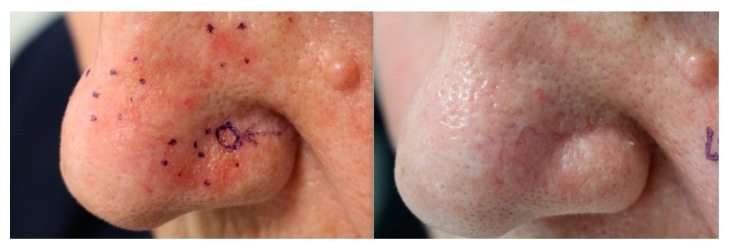
Complete clinical response to photodynamic therapy (PDT), as clearance of actinic keratoses in the nasal area of a patient six weeks after treatment.

As expected, PDT induced a significant reduction in the mean number of AKs per patient, from 7.80 (SD 2.79) to 2.8 (SD 1.61) (*p* = 0.005) (Figure 1). Overall clinical response was complete in 16 patients (64%) and partial in 9 (36%); there were no cases without response.

Histological response was positive in 17 AK (70.8%) and negative in 7 AK (29.2%). Index AK exhibited basal KIN grade 3 in 29.17%, KIN 2 in 41.66%, and KIN 1 in 29.17% of the samples, and after treatment KIN grade was 3 in 8.33%, KIN 2 in 12.50%, KIN 1 in 16.67% and KIN 0 in 62.50% of the lesions, showing a significant improvement of the KIN grade (*p* = 0.004) Considering the KIN grade as a quantitative variable, PDT induced a significant decrease in the mean KIN grade, from 1.88 (SD 0.85) to 0.67 (SD 1.01) (*p* = 0.000).

**Figure 2:**
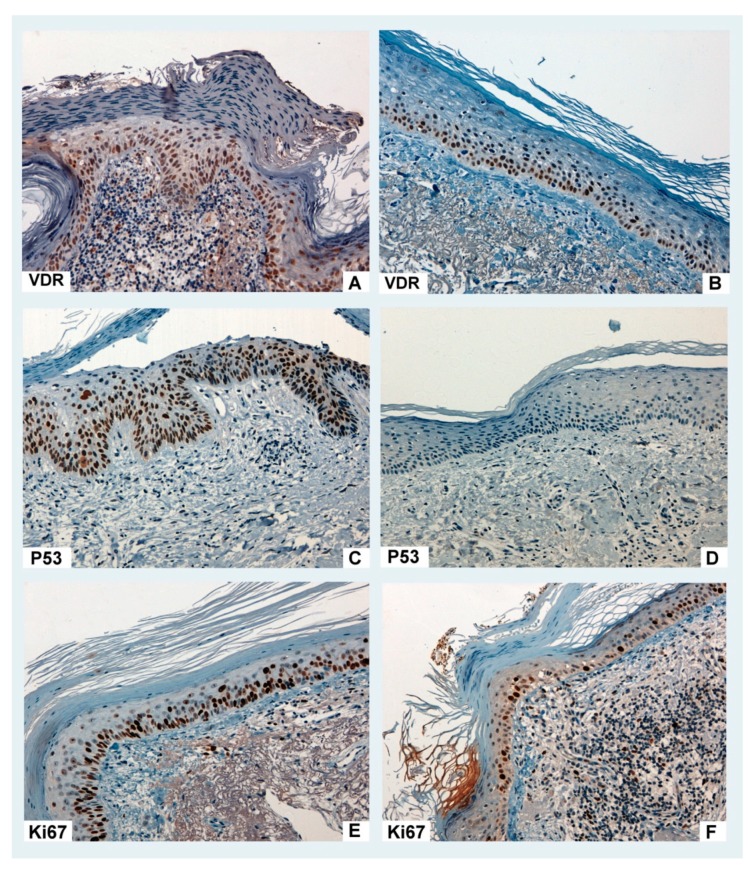
Actinic keratoses: immuno-histochemical response to MAL-PDT (methyl-aminolevulinate photodynamic therapy). Baseline vitamin D receptor (VDR) expression (**A**) did not significantly change after treatment (**B**). Baseline P53 (**C**) and Ki67 (**E**) expression significantly decreased (**D** and **F**, respectively) after PDT.

PDT also induced a significant decrease in the mean of the immunostaining of Ki67 (57.08 (SD 27.10) to 26.88 (SD 19.27), *p* = 0.001) and P53 expression (59.17 (SD 27.72) to 26.39 (SD 24.54), *p* = 0.001). VDR expression increased after PDT but the differences were not statistically significant (56.67 (SD 20.36) to 66.67 (SD 22.00), *p* = 0.062) (Figure 2). No relevant differences were found in the rest of the immunological markers after PDT (Table 2).

### 3.3. Association of Variables with Overall Clinical Response

No statistically significant relationship was found between patient clinical response and age, gender, phototype, AK location and serum 25(OH)D. However, those patients with complete clinical response showed lower VDR expression (35.71, SD 19.88) than those with partial response (62.78, SD 16.74) (*p* = 0.002). Basal β-catenin, Ki67 and P53 expressions were not associated with the overall clinical outcome (Table 3).

### 3.4. Association of the Variables with the Histological Response

No statistically significant relationship was found between age, gender, phototype and location of the AK and the histological response. Patients who responded to PDT had significantly higher serum 25(OH)D levels (26.96 ng/mL, SD 7.49) than those without response (18.60, SD 7.49) (*p* = 0.05). Baseline expression of the explored immunomarkers was not associated with the histological response to PDT (Table 3).

## 4. Discussion

This study supports the relationship between 25(OH)D serum levels and the response of AK to MAL-PDT: VD deficient levels were found to be significantly associated to a lack of response in the reduction of the KIN grade of actinic keratoses, and patients whose AK exhibited a significantly lower VDR basal expression showed a complete clinical response to the treatment. Comparing the histological samples of AK in every patient before and after MAL-PDT, we observed a marginally significant increase in VDR expression after the treatment in addition to the already know reduction in P53 and Ki67 expression [7].

Thus, our findings suggest that a poorer response of AK to MAL-PDT is likely to be expected under a deficient VD status. The mechanisms by which VD may exert an effect on the response of AK to MAL-PDT are unknown. It has been demonstrated that VD promotes UV-induced mutation repair in keratinocytes through an up-regulation of functional P53 [14] and has several antitumoral effects on epidermal neoplasms through the immune system [15,16]. The transcriptional profile of healthy keratinocytes treated with 1,25(OH)D has been studied, showing the up-regulation of some 82 genes and down-regulation of 16 other genes; among those up-regulated were peptidilarginine deaminases, calicreins, serin-protease inhibitors, c-fos or Kruppel-like factor 4, all of which are involved in keratinocyte differentiation [17]. These findings illustrate a poorly understood pro-differentiation network over keratinocytes sustained by VD.

The increase on PpIX accumulation and consequent enhanced tumoral cell death induced by exposure to calcitriol or calcipotriol prior to ALA-PDT has been proposed as a possible mechanism in several previously published studies on murine models [8,9,10]. However, Bay et al. [18] exposed hairless mice to carcinogenic doses of UV-radiation, and found that pre-treatment with calcipotriol prior to MAL-PDT neither increased PpIX accumulation, as measured by fluorescence, nor delayed the onset of SCC compared to MAL-PDT without pre-treatment. Accordingly, VD ability to enhance AK response to PDT may not be related to an increased PpIX accumulation in keratinocytes, but to other mechanisms, perhaps involving its complex effects on keratinocyte proliferation and differentiation.

The relationship between 25(OH)D serum levels and VDR expression in keratinocytes has not been studied in humans. In swine models fed with a VD-deficient diet, VD insufficiency status resulted in a diffuse presence of VDR in the keratinocytic cytoplasm, whilst supplementation with VD up to serum levels of 25(OH)D sufficiency induced a preferentially nuclear location of the VDR [19]. Moreover, unpublished data of our group in several in vitro experiences with skin and vulvar squamous cell carcinoma cell lines (SCC-13 and A-431) reveal that these tumor cells exhibit a diffuse expression of VDR in the cytoplasm that changes dramatically to a predominantly nuclear expression upon the addition of calcitriol to the cell cultures.

As for the discrepancy between the influence of variables on clinical and on histological responses of AK to PDT, it has already been proven that clinical and histological classifications of AK do not accurately match and that conclusions must not be drawn about the histology of AK lesions from their clinical appearance [20]. Hence, we can infer that parallel findings should not necessarily be expected between clinical and histological approaches.

According to our results comparing immunohystochemical features of our samples before and after the treatment, MAL-PDT might be able to increase VDR expression in the nucleus of the keratinocytes in AK. This may improve keratinocyte sensitivity to serum VD, thus providing an additional plausible way through which PDT and VD interact to generate a synergic antitumoral effect on keratinocytic neoplasms such as AK.

An important limitation of this is study is the small sample. A larger sample is warranted to strengthen the evidence provided by our findings.

In summary, our findings imply that serum VD could be considered a modulator of the response of AK to MAL-PDT. Determination of serum 25(OH)D might be appropriate in patients with AK eligible for treatment with MAL-PDT, in order to predict their intrinsic ability to respond to it, and to select those patients who could benefit from VD supplementation prior to treatment. More research is needed, firstly to confirm our results and secondly to establish if VD supplementation in deficient patients previous to PDT might improve its efficacy.

## Figures and Tables

**Table 1:** Sociodemographic and biochemical variables of the sample. (SD: standard deviation; BMI: body mass index.).

Variables (*N* = 25)		Frecuency	Mean (Range or SD)
**Age (years)**			70.1 (61–81)
Gender	Male Female	19/25 (76%) 6/25 (24%)	
Phototype	II III	10/25 (40%) 15/25 (60%)	
B.M.I. (kg/m^2^)			30.1 (23.30–42.40)
Location of treated AK	Face Scalp	9/25 (36%) 16/25 (64%)	
Serum 25(OH)D_3_ (ng/mL)			25.37 (SD 9.86)

**Table 2:** Clinical, histological and immuno-histochemical variables of the sample, before and after MAL-PDT (methyl-aminolevulinate photodynamic therapy).

*N* = 24	Basal (mean, SD)	After PDT (mean, SD)	*p*
**Clinical and histological variables**			
AK number per patient	7.84 (SD 2.79)	2.80 (SD 1.61)	0.005
KIN grade (quantitative)	1.88 (0.85)	0.67 (1.01)	<0.001
KIN grade (qualitative)			0.004
KIN 3	7 (29.17 %)	2 (8.33%)	
KIN 2	10 (41.66%)	3 (12.50%)	
KIN 1	7 (29.17%)	4 ( 16.67%)	
KIN 0	0	15 (62.50%)	
**Immunomarkers**			
VDR expression (%)	56.67 (20.36)	66.67 (22.00)	0.062
VDR intensity (0–3)	1.96 (0.81)	2.08 (0.93)	0.479
β-catenin expression (%)	4.17 (5.69)	2.61 (4.59)	0.191
Ki67 expression (%)	57.08 (27.10)	26.88 (19.27)	0.000
P53 expression (%)	59.17 (27.72)	26.39 (24.54)	0.000

SD: Standard deviation; VDR: vitamin D receptor; KIN: keratinocytic intraepithelial neoplasia; AK: actinic keratosis.

**Table 3:** Influence of clinical and histological variables on overall clinical response of patients and histological response of AK to MAL-PDT.

	Patient Clinical Response		*p*	Histological Response		*p*
	Partial Response (mean, SD) *n* = 6	Complete response (mean, SD) *n* = 19		Positive (mean, SD) *n* = 17	Negative (mean, SD) *n* = 7	
Age (mean, SD)	71.47 (6.66)	69.67 (3.20)	0.53	69.88 (6.19)	73.86 (5.37)	0.153
Gender			1			0.608
Male	14 (73.37%)	5 (26.30%)		14 (73.70%)	5 (26.30%)	
Female	5 (83.30%)	1 (16.70%)		3 (60.00%)	2 (40.00%)	
Phototype			0.175			0.356
II	6 (60.00%)	4 (40.00%)		5 (55.60%)	4 (44.4%)	
III	13 (86.70%)	2 (13.30%)		12 (80.00%)	3 (20.00%)	
Location			1			0.352
-Face	7 (77.80%)	2 (22.20%)		7 (87.50%)	1 (12.50%)	
-Scalp	12 (75.00%)	4 (25.00%)		10 (62.50%)	6 (37.50%)	
Vitamin D (ng/ml)	24.42 (9.67)	27.67 (9.86)	0.483	26.96 (9.49)	18.60 (7.49)	0.05
VDR expression (%)	62.78 (16.74)	35.71 (19.88)	0.002	59.41 (18.53)	53.33 (25.03)	0.535
VDR intensity (0**–**3)	2.00 (0.77)	1.71 (0.95)	0.442	2.00 (0.79)	1.83 (0.98)	0.68
β-cat. expression (%)	4.39 (5.62)	3.86 (5.40)	0.832	5.53 (6.06)	17 (2.04)	0.103
Ki67 expression(%)	56.39 (28.12)	51.00 (32.20)	0.683	55.00 (29.42)	59.17 (22.00)	0.756
P53 expression (%)	64.41 (23.51)	59.00 (38.79)	0.701	64.69 (27.35)	52.00 (22.80)	0.361
